# Hyperhomocysteinemia as a Risk Factor for Vascular Contributions to Cognitive Impairment and Dementia

**DOI:** 10.3389/fnagi.2018.00350

**Published:** 2018-10-31

**Authors:** Brittani R. Price, Donna M. Wilcock, Erica M. Weekman

**Affiliations:** Department of Physiology, Sanders-Brown Center on Aging, University of Kentucky, Lexington, KY, United States

**Keywords:** hyperhomocysteinemia, vascular cognitive impairment and dementia, B vitamins, homocysteine, dementia

## Abstract

Behind only Alzheimer’s disease, vascular contributions to cognitive impairment and dementia (VCID) is the second most common cause of dementia, affecting roughly 10–40% of dementia patients. While there is no cure for VCID, several risk factors for VCID, such as diabetes, hypertension, and stroke, have been identified. Elevated plasma levels of homocysteine, termed hyperhomocysteinemia (HHcy), are a major, yet underrecognized, risk factor for VCID. B vitamin deficiency, which is the most common cause of HHcy, is common in the elderly. With B vitamin supplementation being a relatively safe and inexpensive therapeutic, the treatment of HHcy-induced VCID would seem straightforward; however, preclinical and clinical data shows it is not. Clinical trials using B vitamin supplementation have shown conflicting results about the benefits of lowering homocysteine and issues have arisen over proper study design within the trials. Studies using cell culture and animal models have proposed several mechanisms for homocysteine-induced cognitive decline, providing other targets for therapeutics. For this review, we will focus on HHcy as a risk factor for VCID, specifically, the different mechanisms proposed for homocysteine-induced cognitive decline and the clinical trials aimed at lowering plasma homocysteine.

## Introduction

Vascular contributions to cognitive impairment and dementia (VCID) are defined as the conditions arising from vascular brain injuries that induce significant changes to memory, thinking, and behavior. It is the leading cause of dementia behind only Alzheimer’s disease (AD); however, there is increasing awareness of the co-morbidity of VCID and AD (Bowler et al., 1998; Zekry et al., 2002; Langa et al., 2004; Jellinger and Attems, 2010). Roughly 60% of AD patients have VCID, and it is thought that vascular injuries act as an extra “hit” to the brain that lowers the threshold for cognitive impairment in persons with AD pathology (Schneider and Bennett, 2010; Vemuri and Knopman, 2016). Also, it is suggested that patients with both AD pathology and VCID have a shorter time to dementia and their rate of cognitive decline is faster (Schneider and Bennett, 2010; Vemuri and Knopman, 2016). Recent studies have also shown that vascular injury precedes AD pathologies, highlighting a role for the vasculature in AD progression (Canobbio et al., 2015; Janota et al., 2016).

While there is no cure for VCID, several studies have identified risk factors that can be modified to reduce risk of developing VCID. A major, yet underrecognized, modifiable risk factor for VCID is hyperhomocysteinemia (HHcy). Defined as elevated plasma levels of homocysteine, a non-protein forming amino acid, HHcy has been identified as a risk factor for cardiovascular disease, stroke, VCID, and AD (Graham et al., 1997; Bostom et al., 1999; Eikelboom et al., 1999; Beydoun et al., 2014). Studies have shown that serum homocysteine levels are inversely related to cognitive function in patients with dementia and elevated levels are more common among VCID patients than among AD patients (Miller et al., 2002; Clarke et al., 2003). Elevated plasma homocysteine is also associated with hippocampal atrophy, white matter lesions, and lacunar infarcts (Vermeer et al., 2002; Firbank et al., 2010). In the clinic, it is clear that HHcy plays a role in VCID; however, the mechanisms of homocysteine-induced cognitive impairment and the clinical implications of reducing homocysteine remain unclear. This review paper will focus on proposed mechanisms of homocysteine in the brain, and the clinical trials aimed at lowering homocysteine levels.

## Homocysteine Metabolism

Homocysteine is produced in all cells and involved in the metabolism of cysteine and methionine (Selhub, 1999). Normal levels of homocysteine range between 5 and 15 μmol/L. Levels between 15 and 30 μmol/L are considered mild, levels at 30–100 μmol/L are moderate and levels above 100 μmol/L are considered severe HHcy. During normal metabolism, ATP activates methionine to form *S*-adenosylmethionine (SAM). SAM is a methyl donor to several different receptors and forms *S*-adenosylhomocysteine (SAH) as a by-product of this methyl reaction. SAH can then be hydrolyzed to form homocysteine. Homocysteine can also go through two different re-methylation processes to form methionine again. In one pathway, folate is reduced to tetrahydrofolate which is then converted to 5, 10-methylenetetrahydrofolate. Methylenetetrahydrofolate reductase (MTHFR) reduces 5, 10-methylenetetrahydrofate to 5-methyltetrahydrofolate. Finally, 5-methyltetrahydrofolate and the essential cofactor vitamin B12 add a methyl group to homocysteine to form methionine again. In an alternative pathway, betaine–homocysteine *S*-methyltransferase (BHMT) uses betaine synthesized from choline as a methyl group to convert homocysteine back to methionine.

**FIGURE 1:**
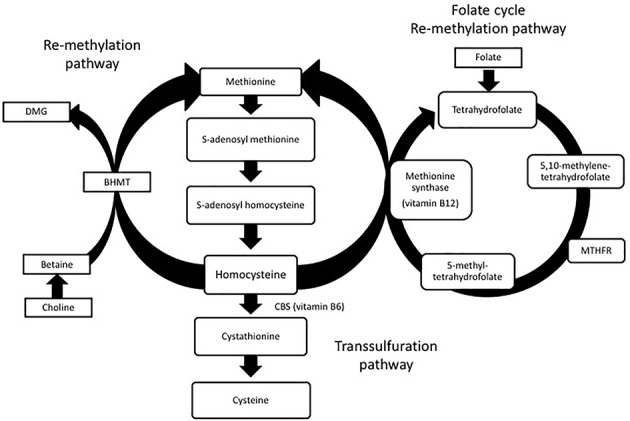
Homocysteine metabolism: homocysteine is converted to methionine or cysteine via remethylation or transsulfuration pathways. Dimethylglycine (DMG), betaine-homocysteine *S*-methyltransferase (BHMT), methylenetetrahydrofolate reductase (MTHFR).

Homocysteine can also go through a transsulfuration pathway to form cysteine. Serine can be enzymatically added to homocysteine by cystathionine beta synthase (CBS) and vitamin B6 to form cystathionine (Locasale, 2013). Cystathionine can then be cleaved by cystathionine gamma lyase (CGL) to form cysteine. While cysteine can be converted back to cystathionine, cystathionine cannot be converted to homocysteine again. The homocysteine metabolic pathway is shown in Figure 1.

## Mechanisms of Homocysteine-Induced Cognitive Impairment

### Posttranslational Modification of Proteins

As mentioned above, homocysteine is produced in all cells; however, its conversion to cysteine or back to methionine does not. The brain lacks both CGL and BHMT, making it dependent on the folate cycle for re-methylation of homocysteine to methionine (Sunden et al., 1997). While this makes the brain especially vulnerable to raised levels of homocysteine, the mechanisms of homocysteine toxicity in the brain remain unclear, with several different mechanisms proposed. Some studies suggest the post-translational modification of proteins by homocysteine, termed homocysteinylation, contributes to its toxicity, especially since the degree of homocysteinylation is proportional to increased level of plasma homocysteine (Jakubowski, 1999; Jakubowski et al., 2000; Perla-Kajan et al., 2007). In the presence of adenosine triphosphate, methionyl-tRNA synthase catalyzes the conversion of homocysteine to homocysteine-thiolactone, which has been shown to homocysteinylate proteins and alter their functions. Specifically, homocysteine thiolactone acts as a Na/K ATPase inhibitor in the hippocampus and cortex of rat brain cells, thus changing the membrane potential of neurons (Rasic-Markovic et al., 2009).

### Oxidative Stress

Other studies suggest homocysteine induces cellular damage via oxidative stress. As mentioned above, during normal homocysteine metabolism, cysteine is produced. Cysteine is a precursor for glutathione, which is a tripeptide that ultimately reduces reactive oxygen species. Without homocysteine conversion to cysteine, either due to CBS mutations or a diet lacking in vitamin B6, glutathione levels decrease, leading to increased reactive oxygen species and ultimately oxidative stress. Homocysteine metabolism is also regulated by the redox potential in a cell since several enzymes involved in its metabolism are regulated by the oxidative status (Zou and Banerjee, 2005). In one instance, the activity of methionine synthase is lowered when reactive oxygen species are high (Zou and Banerjee, 2005). Studies have also shown an increase in neurodegeneration due to homocysteine-related oxidative stress. In cultured embryonic cortical neurons and differentiated SH-SY-5Y human neuroblastoma cells grown in folate free media, there was an increase in cytosolic calcium, reactive oxygen species, and apoptosis (Ho et al., 2003). A significant increase in homocysteine was also found and inhibiting formation of homocysteine prevented the increase in reactive oxygen species. The increase in reactive oxygen species due to HHcy also alters smooth muscle function and promotes proliferation of smooth muscles cells (Welch and Loscalzo, 1998). Homocysteine has also been shown to inhibit endothelial nitric oxide synthase (eNOS) activity in cultured aortic endothelial cells from adult mice (Jiang et al., 2005) and humans (Jiang et al., 2005). In a genetic mouse model of HHcy where the CBS gene is absent, homozygote knockout mice show reduced eNOS activity compared to wildtype mice (Jiang et al., 2005). While the decreased activity of eNOS can affect oxidative stress, it also inhibits endothelial-dependent vasodilation. Taken together with the changes in vascular smooth muscle cells, these data provide further insight into how homocysteine is a risk factor for VCID.

### AMPA and NMDA Receptors

Another proposed mechanism for homocysteine neurodegeneration involves homocysteine’s role as an agonist for AMPA (both metabotropic and ionotropic) and NMDA receptors. Homocysteic acid, an oxidative product of homocysteine that is released in response to excitatory stimulation, acts an excitatory neurotransmitter by activating the NMDA receptor (Cuenod et al., 1990). Activation of both AMPA and NMDA receptors leads to increased intracellular calcium, which in turn leads to activation of several kinases (Robert et al., 2005). Overstimulation of these receptors due to HHcy can then lead to increased free radicals and caspases, which leads to apoptosis (Mattson and Shea, 2003) and neurodegeneration. Using an NMDA antagonist can block the neurotoxic effects of homocysteic acid in the brain (Olney et al., 1987).

### Cerebrovascular

The study of animal models has also lent insight into the mechanisms of homocysteine toxicity and its role in VCID. Several animal models have shown that high plasma levels of homocysteine are sufficient to cause cognitive deficits and vascular adverse events in the brain. Induction of HHcy in an animal model can be achieved via genetic manipulation or diet. Genetic manipulation of either CBS or MTHFR can produce mouse models of HHcy. In humans, deficiencies in CBS result in elevated plasma levels of homocysteine and thrombosis and are the most common cause of hereditary HHcy. CBS^±^ heterozygote mice have a 50% lower CBS activity compared to wildtype mice and develop mild HHcy (Watanabe et al., 1995). These mice show endothelial damage, thickened cerebral arteriolar walls, mild hypertension, and blood–brain barrier dysfunction (Baumbach et al., 2002; Weiss et al., 2003; Kamath et al., 2006). In humans, there are several polymorphisms in MTHFR that produce HHcy and neurological conditions such as a progressive demyelinating neuropathy and cognitive impairment (Clayton et al., 1986; Hyland et al., 1988; Surtees et al., 1991). Chen et al. (2001) deleted the MTHFR gene to create a mouse model of HHcy that exhibits motor and gait abnormalities within 5 weeks after birth. MTHFR^-/-^ homozygotes also present with some loss of function in cerebral vessels and abnormal lipid deposition in the aorta and disruption of the laminar structure of the cerebellum with no obvious changes in the cortex or cerebrum (Neves et al., 2004).

Unlike MTHFR and CBS knockout mice, dietary induction of HHcy allows for age related HHcy to be studied. Dietary induction of HHcy in mice and rats can be achieved through a reduction in the essential cofactors needed for homocysteine conversion (folate, vitamins B6, and B12) or enrichment in methionine, which increases the conversion of methionine to homocysteine. A combination of these diets or even a diet of increased homocysteine can also be used to induce HHcy. Troen et al. (2008) showed that feeding mice a B vitamin deficient diet resulted in cognitive impairment on the Morris water maze and rarefaction of brain capillaries. In another animal model, 6-month-old Sprague–Dawley rats were placed on a diet deficient in folate for 8 weeks. By the end of the 8 weeks, the rats on the folate deficient diet had increased homocysteine levels, ultrastructural changes to cerebral capillaries, endothelial damage, swelling of pericytes, basement membrane thickening, and fibrosis (Kim et al., 2002). Cognitive impairments, decreased acetylcholine in the brain and microhemorrhages were seen in rats that were fed a diet high in homocysteine for 5 or 15 months (Pirchl et al., 2010).

Our lab has also recently developed a model of VCID by inducing HHcy in order to investigate the mechanisms of homocysteine-induced cognitive impairment. We placed 3-month-old C57BL6 mice on a combination diet that is deficient in folate and vitamins B6 and B12 and enriched in methionine (Sudduth et al., 2013) for 3 months. At the end of the 3 months, plasma homocysteine levels reached moderate levels in the mice on the homocysteine diet (82.93 ± 3.561 μmol/L compared to 5.89 ± 0.385 μmol/L in the control mice). When tested on the radial arm water maze for behavioral deficits, these mice exhibited significant cognitive impairments in spatial memory. Prussian blue staining and magnetic resonance imaging showed microhemorrhages were the main cerebrovascular pathology induced by the HHcy diet. The mice on the HHcy diet also had an increase in several pro-inflammatory cytokines along with an increase in matrix metalloproteinase 9 (MMP9) activity. MMP9 has been shown to degrade tight junctions, leading to microhemorrhages and dystroglycans, and the pro-inflammatory cytokines, tumor necrosis factor alpha (TNFα), and interleukin 1 beta (IL-1β), stimulate its transcription (Galis et al., 1994; Vecil et al., 2000; Michaluk et al., 2007; Candelario-Jalil et al., 2011; Klein and Bischoff, 2011). Previous studies have also shown homocysteine can induce MMP9 release from mouse cerebral microvessel endothelial cells (Shastry and Tyagi, 2004). Based on this data, another possible mechanism for homocysteine-induced cognitive impairment could be the pro-inflammatory mediated increase in MMP9 leading to tight junction degradation, microhemorrhages, and, finally, cognitive impairment.

### Astrocytes

In addition to the pathologies listed above, we have also shown that astrocytic end-feet are disrupted in the mice on the HHcy diet (Sudduth et al., 2017). In the brain, astrocytes make up 50% of the cells and their processes, termed astrocytic end-feet, sheath arterioles, and capillaries. The main function of astrocytic end-feet is to maintain ionic and osmotic homeostasis in the brain (Simard and Nedergaard, 2004). To do this, astrocytes have aquaporin four water channels and several potassium channels located at their end-feet. In our mice on the HHcy diet, we found a significant decrease in these channels, as well as other structural markers located at the end-foot. These decreases in the end-foot channels occur after 10 weeks on the HHcy diet. Cognitive deficits and microhemorrhages are also seen starting at 10 weeks on diet. Interestingly, increases in the pro-inflammatory cytokines, TNFα, and IL-1 β, occur after only 6 weeks on diet. We had also previously shown that MMP9 was significantly increased in mice on the HHcy diet (Sudduth et al., 2013). Taken together, we hypothesize that another mechanism of homocysteine-induced cognitive impairment involves the inflammatory-MMP9 pathway. In our hypothesis, homocysteine increases TNFα and IL-1β expression, which in turn activates MMP9, which degrades dystroglycans, a key structural component that anchors the astrocytic end-foot to the basal lamina of the vessels. This disruption of the astrocytic end-foot leads to impaired ionic and osmotic buffering and eventual cognitive impairment.

While several mechanisms of homocysteine-induced cognitive impairment and neurodegeneration have been proposed and discussed here, it is unlikely that homocysteine acts through only one of these mechanisms. Homocysteine may act through several, if not all of these mechanisms. It is also unclear whether the high levels of homocysteine or the lack of B vitamins is the main cause behind the cognitive impairment seen in hyperhomocysteinemic patients. Discussed next are the clinical implications of HHcy and the potential therapeutics tested in clinical trials to lower homocysteine levels and improve cognition.

## Hyperhomocysteinemia in the Clinical Setting

Extensive clinical data support the role of HHcy as a risk factor for VCID. Given that normal and abnormal values are set by individual clinical laboratories, mild-moderate HHcy is loosely defined by clinical standards (Moll and Varga, 2015). However, plasma homocysteine concentrations ranging from 15 and 100 μmol/L are uniformly considered to be indicative of clinically relevant HHcy. Gibson et al. (1964) reported vascular anomalies in patients with homocystinuria (elevated concentration of homocysteine in both plasma and urine), and McCully (1969) introduced his homocysteine hypothesis which connected HHcy with an increased risk of atherosclerosis (Abraham and Cho, 2010). To date, HHcy continues to serve as a widely recognized risk factor for coronary artery disease (CAD), peripheral vascular disease, myocardial infarction (MI), and cerebrovascular disease (CVD; Maron and Loscalzo, 2009). Of particular importance here is the association between HHcy and CVD. CVD can manifest as a stroke, white matter disease, cerebral large vessel disease (atherosclerosis), and cerebral small vessel disease (arteriosclerosis), all of which can independently induce cognitive impairment ranging from subtle deficits to frank dementia (Troen et al., 2008; Maron and Loscalzo, 2009; Hainsworth et al., 2016). Furthermore, HHcy has been associated with hippocampal and white matter atrophy in older subjects with mild hypertension, as well as an increased rate of hippocampal atrophy and cognitive decline in elderly patients (Clarke et al., 1998; Firbank et al., 2010). As suggested by the variety of cellular actions of homocysteine described above, there is no shortage of candidate mechanisms by which HHcy induces cognitive impairment despite known etiologies.

### Hyperhomocysteinemia vs. Homocystinuria

Both genetic mutations and dietary vitamin deficiencies can affect homocysteine levels resulting in HHcy. Several polymorphisms (notably C677T and A1298C) have been identified in the MTHFR gene in humans, which can induce severe HHcy (>100 μmol/L, termed homocystinuria) by limiting conversion of homocysteine back to methionine (Moll and Varga, 2015; Hainsworth et al., 2016). While rare, these polymorphisms induce progressive demyelinating neuropathy and cognitive impairment (Clayton et al., 1986; Hyland et al., 1988; Surtees et al., 1991). That being said, deficiencies in CBS, the rate-limiting enzyme of the aforementioned transsulfuration pathway, are the most common cause of homocystinuria and may result in thrombosis and low levels of cysteine (Sacharow et al., 1993). In contrast to HHcy, homocystinuria is a rare autosomal recessive metabolic disorder characterized by severely elevated plasma homocysteine and subsequently elevated urine homocysteine concentrations. Patients suffering homocystinuria present with developmental delay, osteoporosis, ocular abnormalities, thromoemobolic disease, and severe premature atherosclerosis (Poloni et al., 2018). Given that less marked elevations in plasma homocysteine (i.e., HHcy) are much more common, homocystinuria will not be further discussed in this review. Less marked elevations in plasma homocysteine, referred to as HHcy, may be attributed to factors such as smoking, aging, renal failure, and low dietary levels of folate and vitamins B6 and B12 (Hainsworth et al., 2016).

### Prevalence of B Vitamin Deficiency

As suggested, clinical mild–moderate HHcy is common, especially in elderly patients, with the majority of cases resulting from insufficient B vitamin status (Joosten et al., 1993; Troen et al., 2008). The association of B vitamin status and normal central nervous system function dates back to 1849 when Addison reported on the “wandering mind” of patients with pernicious anemia (Smith and Refsum, 2016). Reports of insufficient B vitamin status with concomitant induction of HHcy trace back to a landmark report by the Framingham Heart Study in 1993. A cohort of 1041 elderly participants (418 men, 623 women) between the ages of 67 and 96 showed that plasma homocysteine becomes elevated due to dietary deficiencies in B6 and folic acid and decreased absorption of B12 (Selhub, 2006; McCully, 2007).

According to the Framingham report, daily intake of 3 mg vitamin B6 and 400 μg of folic acid are required to prevent elevations in plasma homocysteine concentration (Selhub, 2006; McCully, 2007). In support of these amounts of dietary B vitamins, the Nurses’ Health Study revealed that similar levels of dietary B6 and folic acid prevent mortality and morbidity from heart disease (Rimm et al., 1998; McCully, 2007). In the United States, mandatory fortification of grains with folic acid was authorized in 1996 and fully implemented in 1998 (Crider et al., 2011). Prior to fortification of grain products, intakes of B6 and folic acid were well below the recommended quantities (McCully, 2007). By contrast, with the exception of those partaking in a vegan diet, vitamin B12 intake is typically adequate. However, in those >65 years of age lack of gastric acidity, decreased intrinsic factor synthesis by gastric mucosal cells, and history or presence of *H. pylori* infection may contribute to inadequate B12 absorption (McCully, 2007). Not to mention, the aging process itself is associated with decreased ability to absorb B vitamins, which can lead to a gradually rising plasma homocysteine concentration (estimated at 1 μmol/L/decade) (McCully, 2007). Literature now suggests between 5 and 30% of the general population, and 25% of those with vascular diseases, to be affected by HHcy (Selhub, 2006; Peng et al., 2015; Yeh et al., 2016). Granted, because blood homocysteine panels are generally ordered only when patients experience a MI or stroke without traditional risk factors, the aforementioned prevalence of HHcy in the general population is likely skewed and possibly underestimated.

### HHcy, B Vitamin Status, and Cognition

Regardless, public significance of HHcy in the elderly population should not be ignored given that it is easily treatable with B vitamin fortification and serves as a modifiable risk factor for development of cognitive decline, dementia, and AD. A number of early cross-sectional studies relating HHcy or insufficient B vitamin status to cognitive impairment led to generation of the hypotheses suggesting a causal link. In effort to address whether the hypothesis that HHcy induces cognitive impairment is correct, a number of clinical trials have assessed B vitamin refortification with cognitive endpoints. These vitamin refortification trials are outlined in Table 1. Additionally, several meta-analyses of these intervention trials have been conducted (Wald et al., 2010; Ford and Almeida, 2012; Clarke et al., 2014). Upon review, the general consensus suggests that homocysteine-lowering by B vitamin refortification has no significant effect on individual or global cognitive domains despite three trials (FACIT, WAFACS, VITACOG) supporting a beneficial effect. However, when interpreting the results of these trials one needs to consider the fact that many were compromised by the challenges of performing a cognitive clinical trial (cohort age, B vitamin status of said cohort, trial duration, statistical power, etc.).

**Table 1:** B vitamin clinical trials.

Reference	Sample size	Mean participant age (years)	Selection criteria	Intervention	Duration of intervention (years)	Cognitive domains assessed	Additional Study Endpoints	Study Conclusion	Study Limitations
Lewerin et al., 2005	*n* = 209; 126 intervention, 69 placebo	76	Community-dwelling men and women	Placebo vs. 500 μg B12, 800 μg folic acid, 3 mg B6	0.3	Short-term memory, long-term memory, perceptual speed, verbal ability, spatial ability, inductive reasoning	Plasma [Hcy], postural-locomotor-manual (PLM) test	Despite normalization of plasma [Hcy], B vitamin supplementation did not improve cognitive performance.	No reported, significant decline in cognition observed in participants randomized to the placebo arm.
Eussen et al., 2006	*n* = 195; 65 intervention, 65 placebo	82-83	Patients with suspected, mild B12 deficiency	Placebo vs. 1000 μg B12 vs. 1000 μg B12, 400 μg folic acid	0.5	Attention, construction, sensorimotor speed, short-term memory, executive function	Plasma [Hcy]	Supplementation with B12 alone or in combination with folic acid does not improve cognitive performance.	No reported, significant decline in cognition observed in participants randomized to the placebo arm.
Stott et al., 2005	*n* = 185; 23–24/group	72–76	Patients with ischemic vascular disease	Placebo vs. 400 μg B12, 2.5 mg folic acid, 25 mg B6, 25 mg B2 (alone and in various combinations)	1	TICS-M, Information processing	Plasma [Hcy]	Despite normalization of plasma [Hcy], B vitamin supplementation did not improve cognitive performance.	No reported, significant decline in cognition observed in participants randomized to the placebo arm.
VISP Trial Toole et al., 2004	*n* = 3680; 1853 low-dose intervention, 1827 high-dose intervention	66	Patients with history of non-disabling cerebral infarction	Low-dose: 6 μg B12, 20 μg folic acid, 0.2 mg B6 vs. high-dose: 400 μg B12, 2.5 mg folic acid, 25 mg B6	1.8	MMSE	Plasma [Hcy], recurrent cerebral infarction, CHD, death	Despite normalization of plasma [Hcy] in both the low-dose and high-dose intervention groups, supplementation did not improve cognitive performance.	No reported, significant decline in cognition observed in participants randomized to the low-dose intervention.
VITACOG trial Smith et al., 2010; de Jager et al., 2012	*n* = 168; 85 intervention, 83 placebo	76–77	Patients > 70 years with evident MCI (MCI = TICS-M 17-29/30)	Placebo vs. oral TrioBe Plus^®^ containing 0.8 mg folic acid, 0.5 mg B12, 20 mg B6	2	Episodic memory, semantic memory, global cognition (MMSE)	Plamsa [Hcy], Rate of brain atrophy	Sevenfold less regional atrophy in intervention group vs. placebo group. However, this effect was significant only in those with elevated Hcy above the median (i.e., >11.3 μmol.L). Individuals with baseline plasma Hcy above the median, cognitive decline was prevented across all domains assessed.	VITACOG was not powered to detect any effect of plasma [Hcy] on cognition.
McMahon et al., 2006	n = 253; 127 intervention, 126 placebo	73–74	Patients with plasma [Hcy] > 13 μmol/L but normal creatinine concentrations	Placebo vs. 500 μg B12, 1000 μg folic acid, 10 mg B6	2.2	Short-term memory, Learning capacity, Verbal fluency, Semantic fluency, Information processing, Reasoning ability	Plasma [Hcy], Plasma [creatinine]	Despite normalization of plasma [Hcy], B vitamin supplementation did not improve cognitive performance. In fact, information processing speed was improved in those randomized to the placebo arm.	No reported, significant decline in cognition observed in participants randomized to the placebo arm.
FACIT Trial Durga et al., 2007	n = 818; 405 intervention, 413 placebo	60	Patients with plasma [Hcy] > 13 and <26 μmol/L	Placebo vs. 800 μg folic acid	3	Memory, Sensorimotor speed, Complex speed, Information processing, Verbal fluency	N/A	Folic acid supplementation improved subdomains of cognitive function that typically decline with age.	N/A
Vitatops Trial Study Group, 2010	*n* = 8164; 4089 intervention, 4075 placebo	62–63	Patients with recent stroke or TIA	Placebo vs. 0.5 mg B12, 2 mg folic acid, 25 mg B6	3.2	MMSE	Plasma [Hcy], stroke, MI, vascular death	Despite normalization of plasma [Hcy], B vitamin supplementation did not improve global cognitive performance.	Baseline cognitive scores not provided.
NORVIT Trial Bonaa et al., 2006	*n* = 3749; 937 folic acid, B12, B6, 935 folic acid and B12, 934 plus B6, 943 placebo	62–63	Patients ages 30–85 years exhibiting acute MI 7 days before randomization.	Placebo vs. 40 mg B6 vs. 0.8 mg folic acid, 0.4 mg B12 vs. 0.8 mg folic acid, 0.4 mg B12, 40 mg B16 (latter referred to as “combination therapy)	3.3	None	Death, MI, unstable angina pectoris requiring hospitalization, coronary revascularization with percutaneous coronary intervention or coronary artery bypass grafting, and stroke	Despite normalization of plasma [Hcy], B vitamin supplementation did not reduce the risk of recurrent CVD following acute MI. A harmful effect from combined B vitamin supplementation was suggested.	Cognition was not assessed.
SU.FOL.OM3 Trial Andreeva et al., 2011	*n* = 1748; 884 intervention, 864 control (no supplementation)	61	Patients with vascular disease	Placebo vs. 20 μg B12, 560 μg CH3-THF, 3 mg B6 ± 600 mg 2:1 ratio of eicosapentaenoic and docosahexaenoic acid	4.5	TICS-M	Plasma [Hcy]	Despite normalization of plasma [Hcy], B vitamin supplementation did not improve global cognitive performance. However, a possible effect of B vitamin supplementation on cognitive function in those with history of stroke was noted.	Baseline cognitive scores not provided.
HOPE-2 Trial Lonn et al., 2006	*n* = 5522; 2758 intervention, 2764 placebo	69	Patients with history of vascular disease or with history of diabetes and atherosclerosis	Placebo vs. 1 mg B12, 2.5 mg folic acid, 50 mg B6	4.8	MMSE	Plasma [Hcy], death resulting from CVD, MI or stroke	Despite normalization of plasma [Hcy], B vitamin supplementation did not improve global cognitive performance.	No reported, significant decline in cognition observed in participants randomized to the placebo arm.
WAFACS Trial ^∗^Sub-study of HOPE-2 Trial^∗^ Kang et al., 2008	*n* = 2009; 1002 intervention, 1007 placebo	71	Patients with CVD or 3 or more coronary risk factors	Placebo vs. 1 mg B12, 2.5 mg folic acid, 50 mg B6	6	TICS	N/A	B vitamin supplementation failed to delay cognitive decline among patients with CVD or CVD risk factors.	No reported, significant decline in cognition observed in participants randomized to the placebo arm.
Study of the Effectiveness of Additional Reductions in Cholesterol and Homocysteine (SEARCH) Collaborative Group et al., 2010	*n* = 12064; 6033 interventions, 6031 placebo	64	Patients with history of MI	Placebo + 20 mg or 80 mg simvastatin vs. 1 mg B12, 2 mg folic acid + 20mg or 80 mg simvastatin	7.1	TICS-M, verbal fluency test	Plasma [Hcy], major coronary events, stroke, noncoronary revascularizations, death attributable to vascular or non-vascular causes	Despite normalization of plasma [Hcy], B vitamin supplementation did not improve global cognitive performance.	Baseline cognitive scores not provided.

*CH3-THF, methyltetrahydrofolate; CHF, congestive heart failure; CVD, cardiovascular disease; Hcy, homocysteine; MI, myocardial infarction; MMSE, mini-mental state examination; TIA, transient ischemic attack; TICS, telephone interview for cognitive status; TICS-M, modified telephone interview for cognitive status.*

### Limitations of Clinical Trials

As suggested, a number of factors related to trial design and implementation must be considered. First and foremost, the hypothesis being tested should be considered. Assuming the hypothesis is that homocysteine-lowering supplementation with B vitamins slows and/or prevents cognitive decline, those randomized to the placebo arm of the trial must exhibit cognitive decline. As reviewed in Table 1, as well as the aforementioned meta-analyses, the majority of trials conducted fail to report significant cognitive decline in those randomized to the placebo arm. Meaning these trials are limited to showing only that B vitamin treatment does not worsen cognition. Additionally, the age range of trial participants must be considered. Referring to the hypothesis above, if cognition is a study measure the age of the participants should reflect the timeframe in which cognitive decline and dementia occur. Duration of the intervention must also be considered given that elderly individuals exhibiting normal cognition generally decline only by ∼0.1 points on MMSE each year (Hainsworth et al., 2016; Smith and Refsum, 2016). Thus, duration of the intervention must be sufficient to observe cognitive decline, especially if MMSE is to be used as an assessment tool. Three trials represented in Table 1 and seven of the nine trials examined by Wald et al. (2010) in their meta-analysis were of short duration (<12 months) and therefore too short to identify an effect on cognition. Assessment tools must also be sensitive enough to detect subtle changes over the course of the trial. Collectively, age range of the trial cohort, duration of the intervention, and assessment tools will dictate whether the trial design is sufficient to detect an effect. Finally, the appropriateness of the intervention and whether the chosen cohort is likely to respond to that intervention must be considered. Supplied daily doses of B vitamins should be sufficient to lower plasma homocysteine concentrations by at least 20% (Hainsworth et al., 2016). For example, 1 trial addressed in Table 1 prescribed doses of folic acid (0.2 mg) and vitamin B12 (1 μg) that were too low to influence plasma homocysteine (McMahon et al., 2006; Hainsworth et al., 2016). Furthermore, the baseline B vitamin status of each potential participant must be considered at the time of enrollment. This relates back to a cardinal principle of nutrition in which the relationship between vitamin status and a given outcome follows a sigmoidal curve. For example, if a participant exhibited low levels of vitamin B6, additional B6 intake would likely be beneficial, with the opposite being true if the participant’s B6 intake were already high. Additionally, when at the plateau phase (i.e., adequate B6 intake), additional B6 intake will likely have no effect. Despite having critical implications for clinical trials, this principle is often overlooked. Consideration of the participant’s B vitamin status at the time of enrollment would therefore aid in determining whether they are likely to respond to intervention. As such, trial enrollment should only be open to those with insufficient B vitamin status or elevated plasma homocysteine concentration at baseline. Together, these considerations suggest the conclusion that homocysteine-lowering by B vitamin supplementation has no effect on cognition is premature.

### Beneficial Effects of B Vitamin Supplementation

As previously mentioned, results from three trials (FACIT, WAFACS, and VITACOG) do support a beneficial effect of B vitamin supplementation on cognition. The FACIT trial showed significant effects of B vitamins on cognition in participants with high plasma homocysteine, while the WAFACS trial showed similarly significant effects in those with inadequate B vitamin status (Durga et al., 2007; Kang et al., 2008). Furthermore, the VITACOG trial revealed strong effects of B vitamins on both rates of brain atrophy and cognition in individuals with mild cognitive impairment (MCI; Douaud et al., 2013). Further data analysis revealed the sevenfold reduction in regional brain atrophy to be significant only in those with plasma homocysteine concentrations above the median (>11.3 μmol/L) (Douaud et al., 2013). Results of the VITACOG trial thereby imply a threshold effect of plasma homocysteine on measures of brain atrophy and cognition. A threshold effect of plasma homocysteine is further supported by results of the OPTIMA study in which only plasma homocysteine concentrations >11 μmol/L were associated with an increased rate of atrophy of the medial temporal lobe (Clarke et al., 1998). The threshold concept is further supported by a study showing a plasma homocysteine concentration-dependent increase in the rate of cognitive decline in AD patients (Oulhaj et al., 2010). Jointly, these studies suggest the threshold for effect of plasma homocysteine lies between 10 and 11 μM, which may explain why studies conducted in countries that employ mandatory folic acid fortification do not find associations between plasma homocysteine and cognition. Retrospective analysis of the VITACOG data revealed that the protective effect of B vitamin supplementation on both brain atrophy and cognition only occurred in those participants with adequate omega-3 fatty acid status (Jerneren et al., 2015). Additionally, the beneficial effect of B vitamin supplementation on brain atrophy was observed only in participants not routinely taking aspirin (Smith et al., 2010). Omega-3 fatty acid and aspirin statuses may therefore contribute to the failure of B vitamin trials.

In all, given the challenges faced by previous trials, further B vitamin supplementation trials are needed. New trials will be most successful if they prescribe a full combination supplement (B6, B12, and folic acid) at high dose (i.e., dosage sufficient to reduce plasma homocysteine by 20%) to at-risk age participants with elevated plasma homocysteine or inadequate B vitamin status at baseline, adequate omega-3 fatty acid status at baseline, and who do not routinely take aspirin.

## Conclusion

With the number of people aged over 60 expected to increase worldwide by 1.25 billion by 2050, accounting for 22% of the world’s population, it is crucial to understand the causes of dementia and develop treatments (Prince et al., 2015). Current clinical and preclinical data provide strong evidence that HHcy is a key risk factor for VCID. With B vitamin supplementation being an inexpensive and safe therapeutic possibility, it would seem that treatment of HHcy-induced VCID would allow for some progress in lowering the number of dementia patients. Unfortunately, the mechanisms through which HHcy induces cognitive impairment remain unclear, with several different mechanisms proposed. In addition, clinical trials aimed at lowering homocysteine levels via B vitamin supplementation have also been lacking in their study design and ability to properly test the hypothesis that lowering homocysteine can slow and/or prevent cognitive decline. Future studies involving preclinical animal models and properly designed clinical trials will be necessary in order to effectively treat HHcy-induced VCID and lower the incidence of dementia.

## Author Contributions

BP and EW each wrote 50% of the manuscript. DW edited for content, checked for accuracy, and provided guidance in the preparation of the content.

## Conflict of Interest Statement

The authors declare that the research was conducted in the absence of any commercial or financial relationships that could be construed as a potential conflict of interest.
